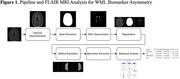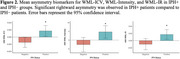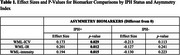# Rightward White Matter Disease is Correlated to Intraplaque Hemorrhage

**DOI:** 10.1002/alz70856_104119

**Published:** 2025-12-26

**Authors:** Faraz Honarvar, Joshua Noronha, Adam Gibicar, Pascal Tyrrell, Pejman Maralani, Corinne Fischer, Sandra E. Black, Alan R. Moody, April Khademi

**Affiliations:** ^1^ Queen's University, School of Medicine, Kingston, ON, Canada; ^2^ McMaster University, Department of Kinesiology, Hamilton, ON, Canada; ^3^ Toronto Metropolitan University, IAMLAB, Department of Electrical, Computer and Biomedical Engineering, Toronto, ON, Canada; ^4^ University of Toronto, Department of Medical Imaging, Toronto, ON, Canada; ^5^ University of Toronto, Institute of Medical Science, Toronto, ON, Canada; ^6^ Keenan Research Center for Biomedical Science, St. Michael's Hospital, Unity Health Network, Toronto, ON, Canada; ^7^ Division of Neurology, Department of Medicine, University of Toronto, Toronto, ON, Canada; ^8^ University of Toronto, Toronto, ON, Canada

## Abstract

**Background:**

Cerebrovascular disease (CVD) is a leading cause of mortality with a strong link to cognitive impairment and dementia. White matter lesions (WML) are prevalent in CVD and are early markers of vascular compromise, particularly in relation to intraplaque hemorrhage (IPH), an indicator of carotid artery plaque instability. As vascular disease represents a possible treatment window for dementia subjects, this study explores the relationship between hemispheric WML asymmetry and IPH utilizing a large multicenter cohort to find novel biomarkers of disease.

**Method:**

FLAIR MRI scans of 264 subjects from the Canadian Atherosclerosis Imaging Network were categorized as IPH positive (IPH+) or IPH negative (IPH‐) and WML biomarkers were automatically computed (Figure 1). Biomarkers related to WML prevalence (volume) and WML ischemia and progression (intensity) were extracted: ICV‐normalized WML volume (WML‐ICV), WML mean intensity (WML‐Intensity), and WML intensity ratio (WML‐IR). WML asymmetry was assessed via an asymmetry index measure (AIM). Linear mixed models and regression analyses were conducted, with adjustments for age, sex, scanner manufacturer, and stenosis, to evaluate associations between WML biomarkers and IPH status.

**Result:**

IPH+ patients exhibited significant rightward asymmetry in WML‐ICV (0.0032 ± 0.002, *p* < 0.05), WML‐Intensity (7.26 ± 5.41, *p* < 0.05), and WML‐IR (0.0271 ± 0.0204, *p* < 0.05); Table 1. IPH+ subjects (left, right or bilateral) had more lesions that were brighter in the right hemisphere. This trend was most pronounced in younger male patients (<65 years), suggesting a high‐risk demographic. Regression analysis revealed IPH as a significant predictor of WML asymmetry, with stronger effects observed in subjects with IPH in the right carotid artery.

**Conclusion:**

Previous studies suggest more injury in the right hemisphere for subjects with small vessel disease, and this work supports this finding. With rightward WML asymmetry being strongly associated with IPH, this could be reflecting a surrogate marker for overall vascular disease and its contribution to brain health and dementia. Automated WML biomarkers can be used to identify these high‐risk patients and guide early interventions for subjects with vascular disease and dementia. Future work should validate these findings in larger, longitudinal datasets to enhance clinical applications.